# Neutrophil-lymphocyte count ratio as a biomarker of severe sepsis in *Escherichia coli *infections in adults

**DOI:** 10.1186/cc11963

**Published:** 2013-03-19

**Authors:** LR Ljungstrom, G Jacobsson, R Andersson

**Affiliations:** 1 Skaraborgs Sjukhus, Skövde, Sweden; 2 Gothenburg University, Gothenburg, Sweden

## Introduction

The neutrophil-lymphocyte count ratio (NLCR) is an easy to analyse biomarker reacting very early in the course of acute inflammation. It has previously been reported to correspond to bacteremia and recently to disease severity in community-acquired pneumonia [1]. We have looked at 205 consecutive patients with *Escherichia coli *infections (ECI) and found the same to be true for ECIs. This may be of great clinical importance since *E. coli *is the most frequently isolated pathogen in patients with infections requiring in hospital care.

## Methods

This study is part of a 9-month consecutive study of community-acquired severe sepsis and septic shock in adults at Skaraborg Hospital in the western region of Sweden. The hospital serves a population of 256,000 inhabitants and has approximately 60,000 annual visits to the ED. All patients admitted to the hospital receiving intravenous antibiotic treatment within the first 48 hours of admission were evaluated for severe sepsis and septic shock. Upon admission, two sets of blood cultures and other relevant cultures were obtained from each patient as well as sampling for NLCR and venous plasma lactate. The patient records were evaluated by one infectious diseases specialist. Approximately 2,300 patients were diagnosed as having a bacterial infection. From those, an informed consent to participate in the study could be obtained from approximately 1,600 patients.

## Results

Of the 1,600 patients who gave consent to participate in the study, 205 had an ECI. Sixty-four had a positive blood culture for *E. coli*. Fifty of the patients met one or more criteria for severe sepsis or septic shock. The NLCR was significantly higher (*P <*0.001) within the severe sepsis group (median = 21.1 with quartiles 11.1 to 42.4) compared with the group with no severe sepsis (median = 11.6 with quartiles 7.6 to 18.9).

## Conclusion

The NLCR can be used as a biomarker of disease severity even in ECIs. The biomarker reacts rapidly, is cheap and needs no extra sampling. The higher the value, the higher the probability for severe sepsis. A high value can even precede the development of severe sepsis or septic shock. However, a low value never excludes neither bacteremia nor severe sepsis. The method cannot be used in patients with disturbances in neutrophil or lymphocyte levels due to other causes than sepsis.
